# Supplementary material for: Self-assessment and rest-activity rhythm monitoring for effective bipolar disorder management: a longitudinal actigraphy study
Source: Int J Bipolar Disord. 2025 Nov 28;13:34. doi: 10.1186/s40345-025-00401-x (PMC12662911; doi:10.1186/s40345-025-00401-x)
Supplement: Supplementary file 1 — Supplementary Material 1. [file 40345_2025_401_MOESM1_ESM.pdf]

SUPPLEMENTARY MATERIAL

To the paper:

Self-assessment and circadian rhythm monitoring for effective bipolar disorder management: a longitudinal actigraphy study, by Bianca Pfaffenseller, Jakub Schneider, Taiane de Azevedo Cardoso, Mario Simjanoski, Martin Alda, Flavio Kapczinski, Eduard Bakstein

1. Convergent validity: ASERT vs YMRS and MADRS clinical scales

The table SM1 shows linear mixed-effect random intercept model results, comparing the ASERT subscores to the respective clinical scale summary scores. ASERT responses were obtained within 7 days from the clinical scale. Significance testing done using the Satterwaite's method (lmerTest R package).

Table S1 Comparison of ASERT subscores to relevant clinical scales.

	**YMRS LME vs ASERT_MAN**			**MADRS LME vs ASERT_DEP**			**MADRS LME vs ASERT_DEPNSP**		
Predictors	*Estimates*	*CI*	*p*	*Estimates*	*CI*	*p*	*Estimates*	*CI*	*p*
(Intercept)	2.64	1.66 – 3.62	<0.001	3.96	1.38 – 6.54	0.003	2.75	0.01 – 5.49	0.049
**ASERT mania**	**0.38**	**0.18 – 0.59**	**<0.001**						
**ASERT depression**				**1.42**	**1.05 – 1.80**	**<0.001**			
**ASERT depression + non-specific**							**1.04**	**0.77 – 1.31**	**<0.001**
**Random Effects**									
σ2	10.23			48.54			47.74		
τ00	1.11 ID			10.94 ID			10.73 ID		
ICC	0.10			0.18			0.18		
N	19 ID			19 ID			19 ID		
Observations	116			116			116		
Marginal R2 / Conditional R2	0.124 / 0.210			0.350 / 0.469			0.371 / 0.487		

2. Internal structure of the ASERT

The table S2 shows the internal structure of the ASERT responses, as analyzed on all 531 ASERT responses.

Table S2. Internal structure of the ASERT questionnaire resulting from the PCA analysis.

	**#**	**Item text**	**PC1**	**PC2**	**PC3**	**PC4**	**PC5**	**PC6**	**PC7**	**PC8**	**PC9**	**PC10**
**depression**	1	I feel sad, downhearted	0.418	-0.130	0.196	-0.311	0.108	-0.286	0.079	-0.612	-0.284	-0.345
	2	I do not enjoy anything, and nothing pleases me	0.371	-0.092	0.275	0.059	-0.043	-0.072	-0.080	-0.203	0.781	0.332
	3	I have no energy	0.396	-0.172	-0.040	0.477	0.278	-0.181	-0.576	0.173	-0.305	0.140
	4	I feel gloomy and pessimistic about the future	0.415	-0.083	0.438	-0.129	-0.406	-0.036	0.267	0.579	-0.200	0.002
**mania**	5	I feel unusually great, optimistic	-0.058	0.449	-0.012	0.270	-0.433	-0.449	-0.231	0.018	0.189	-0.493
	6	I have excess energy	-0.026	0.506	0.098	0.094	-0.202	-0.184	0.117	-0.290	-0.342	0.660
	7	My thinking is very fast, others cannot keep up with me	0.158	0.496	0.045	-0.039	0.688	-0.213	0.318	0.285	0.133	-0.087
	8	I need to sleep less than usual	0.061	0.430	0.399	-0.200	0.056	0.606	-0.467	-0.054	-0.048	-0.138
**non-specific**	9	I feel restless, tense	0.370	0.175	-0.646	-0.545	-0.156	-0.030	-0.228	0.141	0.059	0.131
	10	I cannot focus	0.436	0.140	-0.323	0.484	-0.125	0.480	0.382	-0.178	-0.012	-0.168
		**Standard deviation**	2.352	1.847	0.880	0.773	0.707	0.640	0.612	0.517	0.453	0.423
		**Proportion of Variance**	0.451	0.279	0.063	0.049	0.041	0.033	0.031	0.022	0.017	0.015
		**Cumulative Proportion**	0.451	0.730	0.793	0.842	0.883	0.916	0.947	0.969	0.985	1.000

3. ASERT vs BRIAN and FAST

Tables S3 and S4 provide overview of linear mixed effect model parameters for comparison of the BRIAN and FAST scales (dependent variable) predicted by responses to the *depressive* and *manic* part of the ASERT, respectively.

Table S3.a: ASERT_DEP vs FAST and BRIAN

	FAST.TOTAL			BRIAN.TOTAL			BRIAN.RHYTHM		
Predictors	*Estimates*	*CI*	*p*	*Estimates*	*CI*	*p*	*Estimates*	*CI*	*p*
(Intercept)	14.33	8.48 – 20.18	**<0.001**	31.36	27.89 – 34.83	**<0.001**	6.31	5.74 – 6.88	**<0.001**
sum quest dep	1.18	0.72 – 1.63	**<0.001**	1.20	0.81 – 1.58	**<0.001**	0.01	-0.04 – 0.06	0.695
**Random Effects**									
σ^2	55.07			43.93			0.63		
τ_{00}	134.93 $_{ID}$			35.68 $_{ID}$			1.22 $_{ID}$		
ICC	0.71			0.45			0.66		
N	19 $_{ID}$			19 $_{ID}$			19 $_{ID}$		
Observations	112			113			113		
Marginal R^2 Conditional R^2	/0.102 / 0.740			0.224 / 0.572			0.001 / 0.658		

Table S3.b: ASERT_MAN vs FAST and BRIAN

	FAST.TOTAL			BRIAN.TOTAL			BRIAN.RHYTHM		
Predictors	*Estimates*	*CI*	*p*	*Estimates*	*CI*	*p*	*Estimates*	*CI*	*p*
(Intercept)	22.43	15.93 – 28.93	**<0.001**	37.42	33.48 – 41.37	**<0.001**	6.09	5.58 – 6.60	**<0.001**
sum quest man	-0.98	-1.74 – -0.22	**0.012**	-0.29	-0.93 – 0.36	0.380	0.08	0.01 – 0.16	**0.021**
**Random Effects**									
σ^2	63.88			58.63			0.64		
τ_{00}	163.01 $_{ID}$			44.66 $_{ID}$			0.87 $_{ID}$		
ICC	0.72			0.43			0.58		
N	19 $_{ID}$			19 $_{ID}$			19 $_{ID}$		
Observations	112			113			113		
Marginal R^2 Conditional R^2	/0.044 / 0.731			0.009 / 0.437			0.049 / 0.597		

4. ASERT and clinical scales vs actigraphy

Table S5 summarizes the mixed-effect model results for comparison between actigraphy, ASERT and clinical scales. Beta slope coefficients(β1) are calculated for scaled (z-transformed) predictor data for comparability. The ε^2 are the estimated epsilon squared effect size values.

Table S4: Results of LME models comparing actigraphy to clinical scales and ASERT

predictor	Sleep_duration			Amplitude			Mesor			Acrophase			IS			IV		
**dep. variable**	**β1**	**P**	**ε2**	**β1**	**P**	**ε2**	**β1**	**P**	**ε2**	**β1**	**P**	**ε2**	**β1**	**P**	**ε2**	**β1**	**P**	**ε2**
MADRS	**2.802**	**0.023**	0.107	**-2.604**	**0.039**	0.132	**-3.451**	**0.006**	0.259	2.202	0.073	0.061	**-3.081**	**0.012**	0.153	**3.088**	**0.012**	0.289
YMRS	0.574	0.160	0.014	-0.320	0.436	0.000	-0.314	0.445	0.000	0.683	0.116	0.058	-0.790	0.052	0.038	0.223	0.587	0.000
BRIAN.TOTAL	2.192	0.120	0.029	-2.965	0.053	0.076	**-3.418**	**0.032**	0.112	2.623	0.057	0.051	**-3.858**	**0.005**	0.122	2.791	0.068	0.062
BRIAN.RHYTHM	-0.253	0.066	0.034	-0.120	0.442	0.000	0.055	0.735	0.000	**0.439**	**0.001**	0.167	-0.215	0.109	0.023	-0.037	0.812	0.000
FAST.TOTAL	3.184	0.058	0.036	-3.069	0.104	0.027	-3.673	0.059	0.045	2.608	0.121	0.020	-3.186	0.050	0.039	1.281	0.498	0.000
ASERT_dep	**0.869**	**0.000**	0.035	**-0.905**	**0.002**	0.029	**-1.313**	**0.000**	0.059	**0.979**	**0.000**	0.077	**-0.955**	**0.000**	0.046	0.340	0.205	0.002
ASERT_man	**-0.694**	**0.001**	0.031	**0.615**	**0.011**	0.016	**0.982**	**0.000**	0.044	-0.042	0.858	0.000	0.265	0.178	0.002	-0.267	0.235	0.001

5. ASERT and actigraphy-based predictors of depressive relapse

Table S5 summarizes the ASERT-based and actigraphy-based features, used for depression prediction and resulting principal components of the supervised PCA procedure that were used in the model. Three different models and thus three different PCA components are compared. The features marked _*diff* correspond to differences between feature values from subsequent weeks.

Table S5: Predictive features for depressive relapse prediction

	ASERT only		Actigraphy only		Combined		
**Relevant features**	**PC1**	**PC2**	**PC1**	**PC2**	**PC1**	**PC2**	**PC3**
asert_q_1	0.192	-0.204			0.187	-0.078	-0.175
asert_q_2	0.214	-0.241			0.216	0.030	-0.204
asert_q_3	0.206	-0.221			0.199	-0.101	-0.179
asert_q_4	0.223	-0.190			0.224	0.024	-0.160
asert_q_9	0.153	-0.204			0.152	-0.035	-0.192
asert_q_10	0.206	-0.231			0.202	-0.057	-0.210
asert_fill_time	0.110	0.039			0.102	-0.057	0.067
asert_sum_dep	0.241	-0.246			0.238	-0.038	-0.206
asert_sum_all	0.244	-0.255			0.246	-0.003	-0.246
asert_sum_ns	0.197	-0.239			0.195	-0.051	-0.220
asert_sum_dep_ns	0.247	-0.267			0.244	-0.047	-0.232
asert_q_1_diff	0.230	0.294			0.223	-0.020	0.287
asert_q_2_diff	0.285	0.316			0.278	0.0268	0.323
asert_q_3_diff	0.246	0.187			0.234	-0.088	0.197
asert_q_4_diff	0.271	0.226			0.264	-0.022	0.236
asert_q_10_diff	0.230	0.164			0.225	-0.036	0.148
asert_sum_dep_diff	0.283	0.278			0.274	-0.032	0.284
asert_sum_ns_diff	0.224	0.158			0.223	0.014	0.134
asert_sum_dep_ns_diff	0.282	0.252			0.275	-0.017	0.246
Mesor_14			0.355	-0.317	0.073	0.339	-0.059
IS_14			0.353	0.372	0.002	0.359	0.076
IV_14			-0.396	0.148	-0.067	-0.379	0.098
Ampl_14			0.402	-0.075	0.099	0.383	0.002
SleepDur_MAD_14			-0.151	-0.327	0.030	-0.166	-0.154
Mesor_7			0.354	-0.351	0.077	0.338	-0.088
IS_7			0.335	0.437	-0.001	0.339	0.060
IV_7			-0.396	0.159	-0.057	-0.379	0.129
SleepDur_MAD_7			-0.106	-0.371	0.060	-0.127	-0.143
SleepDur_MAD_7_diff			0.056	0.388	-0.068	0.078	0.162

6. Confusion matrices for depression relapse detection

The tables S6 a, b, and c, provide confusion matrices for the depression-remission classification task. Each table provides the test-set results of the three classifiers, each based on a different set of input features: Classifier using a combination of ASERT self-assessments and actigraphy (S6a), Classifier using only ASERT-based features, (S6b), and a classifier using only actigraphy-based features (S6b). The aggregate measures are provided in the main text.

Table S6a: Model using *combination of actigraphy and ASERT*

	Predicted Class - Remission	Predicted Class - Depression
True Class - Remission	48	2
True Class - Depression	5	10

Table S6b: Model using *ASERT only*

	Predicted Class - Remission	Predicted Class - Depression
True Class - Remission	47	3
True Class - Depression	7	8

Table S6c: Model using *actigraphy only*

	Predicted Class - Remission	Predicted Class - Depression
True Class - Remission	49	1
True Class - Depression	11	4
